# Phenotypically defined subpopulations of circulating follicular helper T cells in common variable immunodeficiency

**DOI:** 10.1002/iid3.326

**Published:** 2020-07-02

**Authors:** Sait Yesillik, Sudhir Gupta

**Affiliations:** ^1^ Division of Basic and Clinical Immunology University of California Irvine California

**Keywords:** autoimmunity, CVID, follicular helper T cells

## Abstract

**Background:**

Common variable immunodeficiency (CVID) is characterized by low immunoglobulin G and IgA/IgM, decreased switched memory B cells, impaired response to vaccine, and an increased susceptibility to infections and autoimmunity. T_FH_ cells play an important role in germinal center reaction where it supports isotype switching, somatic hypermutation, generation of memory B cells, and differentiation of B cells to plasma cells. The objective was to study the distribution of three subsets of T_FH_ cells and their relationship with autoimmune diseases associated with CVID.

**Methods:**

T_FH_ cells have been divided into T_FH_1 (interleukin 21 [IL‐21] and interferon γ), T_FH_2 (IL‐21 and IL‐4), and T_FH_17 (IL‐21 and IL‐17) cells. Mononuclear cells from 25 patients with CVID and age and gender‐matched controls were stained with various monoclonal antibodies (anti‐CD4 APC, anti‐CXCR5 FITC, anti‐CCR6 PerCP, and anti‐CXCR3 PE) and isotype controls and analyzed for T_FH_1 (CD4^+^CXCR5^+^CXCR3^+^CCR6^−^), T_FH_2 (CD4^+^CXCR5^+^CXCR3^−^CCR6^−^), and T_FH_17 (CD4^+^CXCR5^+^CXCR3^−^CCR6^+^) cells by multicolor flow cytometry. Twenty thousand cells were acquired and analyzed by FlowJo software. Statistical analysis of comparison of patients and healthy controls was performed by paired *t* test using PRISM 7 software.

**Results:**

T_FH_2 and T_FH_17 cells subpopulations of T_FH_ cells were significantly decreased (*P* < .003 and *P* < .006, respectively) in CVID as compared with controls. No significant difference was observed in any of T_FH_ cell subpopulations between CVID with and those without autoimmunity group.

**Conclusion:**

Alterations in T_FH_ cell subpopulation may play a role in defects in B cell compartment in CVID.

## Graphical abstract

In this study, we have examined three subsets of follicular helper T cells in common variable immunodeficiency (CVID) and demonstrated their alterations that could explain some of the defects in B cell compartment in CVID.

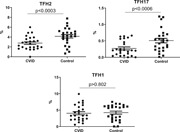

## 1 INTRODUCTION

Common variable immunodeficiency (CVID) is heterogeneous and most common primary immunodeficiency disease in adults characterized by low serum immunoglobulins immunoglobulin G (IgG), IgA, and/or IgM, impaired specific antibody response to vaccines, and increased susceptibility to recurrent infections.^1^, ^5^ In addition, patients with CVID have increased prevalence of allergic, autoimmune, and granulomatous disorders, and malignancy, the majority being lymphoreticular malignancy.^5^, ^10^

A number of gene mutations have been reported in CVID; however, they account for less than 20% of CVID patients.^11^, ^13^ Therefore, in majority of patients with CVID cause(s) is unknown. The predominant defects appear to be in the B cell compartment including impaired immunoglobulin isotype switching and differentiation of B cells into plasma cells despite normal number of B cells; postgerminal center B cells are defective and switched memory B cells are reduced.^14^, ^16^

The follicular helper (T_FH_) cells are major CD4^+^ T helper subset that are essential for B cell differentiation into immunoglobulin producing plasma cells, and for the generation of memory B cells in the germinal center (GC).^17^, ^18^ GCs are primary sites for class‐switched recombination and affinity maturation. T_FH_ cells regulate GC formation, and selection of high‐affinity antibody‐producing B cells and support isotype class switching.^19^, ^20^ An increased cT_FH_ cells response in the GC is associated with the expansion of low affinity and autoreactive B cells.^21^, ^22^

T_FH_ cells are characterized by the expression of CXCR5 and transcription factor B cell lymphoma 6 (Bcl6), and production of their signature cytokine, the interleukin 21 (IL‐21).^23^, ^25^ CXCR5 plays and important role in the migration of B cells to germinal follicles to support immunoglobulin production.^26^ Although T_FH_ cells are predominantly found in lymph nodes and spleen, a small proportion of these cells are also found in the circulation. Vella et al^27^ compared T_FH_ cells from lymph nodes, thoracic duct lymph, and blood and showed that they share TCR clonotype, phenotype, and transcriptional signature, and therefore cT_FH_ represents T_FH_ cells in GC.

Morita et al^28^ also reported that blood CXCR5^+^ CD4^+^ T cells induce naive B cells differentiation and class switching more than CXCR5^−^ CD4^+^ T cells. According to the expression of CXCR3 and CCR6 on CD4 + CXCR5, they identified three different subsets of T_FH_ cells with different functions. In addition to IL‐21, these different cT_FH_ subsets can also produce, albeit in lower amounts, IL‐4, interferon γ (IFN‐γ), and IL‐17. cT_FH_1 (CXCR5^+^CXCR3^+^CCR6^−^) produce IFN‐γ, cT_FH_2 (CXCR5^+^CXCR3^−^CCR6^−^) produce IL‐21 and IL‐4, and cT_FH_17 (CXCR5^+^CXCR3^−^CCR6^+^) produce IL‐21 and IL‐71A; all of them are able to efficiently induce antibody response by memory B cells.

A role of T_FH_ cells in antibody‐mediated autoimmune disease has been established in both mice and humans.^21^, ^22^ Because T_FH_ cells play a role in class switching and autoimmunity, and an observed deficiency of switched memory B cells and increased autoimmunity in CVID, we evaluated cT_FH_1, cT_FH_2, and cT_FH_17 cells in CVID patients and examined their relationship with autoimmune diseases associated with CVID.

## 2 MATERIALS AND METHODS

### 2.1 Subjects

A total of 25 patients (seven men and 18 women, aged 15‐82 years) with CVID and 25 healthy controls (13 men and 12 women, aged, 20‐67 years) were enrolled in the study. Pan American and ESID Criteria were used to diagnose CVID patients.^1^ Clinical and immunological features of these patients have been published.^29^ All patients were receiving immunoglobulin replacement treatment. Blood samples were drawn at trough level. The Institutional Review Board committee (human research), University of California at Irvine approved this study protocol. Written and signed informed consent was obtained from all subjects.

### 2.2 Antibodies

Anti‐CD4 APC, anti‐CXCR5 (CD185) FITC (clone‐2G8), anti‐CCR6 (CD196) PerCP (clone‐11A9), anti‐CXCR3 (CD183) PE (clone‐1C6/CXCR3) monoclonal antibodies, and isotype control antibodies were purchased from Pharmingen BD Sciences, San Jose, CA.

### 2.3 Immunophenotyping

Ten ml of heparinized blood was diluted with Hank's buffered salt solution (HBSS). Mononuclear cells (MNC) were separated by Ficoll‐Hypaque density gradient using lymphocyte separation medium. Cells were suspended in HBSS and used for immunophenotyping. Cells were incubated with different monoclonal antibodies and isotype controls (below) for 30 minutes on ice in the dark. Cells were washed and cT_FH_1, cT_FH_2, and cT_FH_17 analyses were performed by multicolor flow cytometry (FACSCelesta; Becton‐Dickinson, San Jose, CA). Twenty thousand cells were acquired and analyzed by FlowJo software (Treestar Inc., Ashland, OR).

For cT_FH_ cells: anti‐CD4 APC, anti‐CXCR5 FITC, anti‐CCR6 PerCP, anti‐CXCR3 PE; three subsets of cT_FH_ cells were identified as: cT_FH_1 (CD4^+^CXCR3^+^CCR6^−^), cT_FH_2 (CD4^+^CXCR3^−^CCR6^−^), and cT_FH_17 (CD4^+^CXCR3^−^CCR6^+^).

Statistical analysis of comparison of patients and healthy controls was performed by paired *t* test for equality of means using PRISM 7 software.

## 3 RESULTS

### 3.1 cT_FH_ subpopulations in CVID

**Figure 1:**
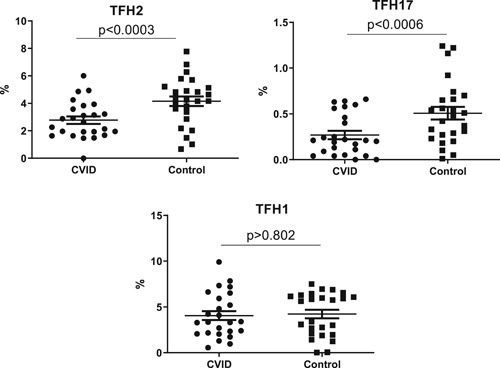
T_FH_ cell subsets in CVID and controls. Mononuclear cells from 25 CVID patients and healthy controls were stained with monoclonal antibodies and isotype. CVID, common variable immunodeficiency

CXCR5 + CD4 cT_FH_ are further subdivided by the expression of CXCR3 and CCR6 and cytokines they produce into T_FH_1, T_FH_2, and T_FH_17 cells.^28^ MNC were incubated with panel of monoclonal antibodies defining T_FH_1, T_FH_2, and T_FH_17 cells and isotype controls and analyzed using multicolor flow cytometry. Cumulative data from 25 patients with CVID and healthy controls are shown in Figure 1. cT_FH_2 and cT_FH_17 cells were significantly decreased in CVID patients when compared to controls (*P* < .003, *P* < .006, respectively). cT_FH_1 cells were comparable between two groups (*P* < .802).

controls to defined T_FH_1, T_FH_2, and T_FH_17 subsets of follicular helper T cells and analyzed with multicolor flow cytometry using FACSCelesta. Data are expressed as mean ± SD. Statistical analysis was performed with GraphPad Prism version 8.4.3 for Windows (GraphPad Software, San Diego, CA).

### 3.2 cT_FH_ subpopulations in CVID with and without autoimmunity

**Figure 2:**
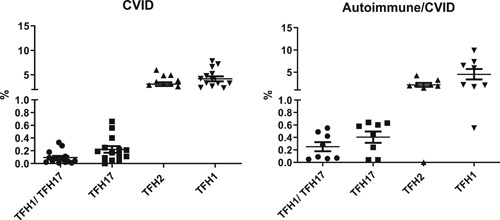
T_FH_ cell subsets relations to autoimmune diseases in CVID. T_FH_1, T_FH_2, and T_FH_17 subsets and T_FH_1/T_FH_17 ratio were compared for CVID patients with autoimmune diseases (*n* = 7) and without autoimmune diseases (*n* = 18). CVID, common variable immunodeficiency

cT_FH_ cells play a role in autoimmunity and autoimmune diseases.^21^, ^22^, ^30^ Therefore, we analyzed our data for the presence and absence of autoimmunity in CVID. Data are shown in Figure 2. cT_FH_17 cells tended to be higher in CVID patients with autoimmunity as compared with those without autoimmunity. However, we observed no significant difference in cT_FH_1, cT_FH_2, and cT_FH_17 cells between CVID patients with or without autoimmune disease (*P* > .754, *P* > .177, *P* > .230, respectively). There were only seven of 25 CVID patients with autoimmune disease.

## 4 DISCUSSION

Patients with CVID display increased susceptibility to recurrent infections, and increased incidence of autoimmune and inflammatory disorders, and malignancy.^2^, ^10^ The hallmark of defect In CVID is an impaired specific antibody response to vaccine, decreased switched memory B cells, and impaired differentiation of B cells to plasma cells that takes place in GCs of follicles.^14^, ^16^

T_FH_ cells are specialized helper T cells that provide help to B cells and are essential for the formation of GC B cells, affinity maturation, and generation of high‐affinity antibodies and memory B cells. T_FH_ cells.^17^, ^28^, ^31^ T_FH_ cells are characterized by high expression of the transcription factor Bcl6, CXCR5, and IL‐21 production.^24^, ^26^ The GC is also regulated by T follicular regulatory cells.^17^

CVID is the most common and genetically heterogeneous antibody deficiency disorder in adults. However, with the use of genome‐wide association studies and next‐generation sequencing have delineated several gene mutations in CVID including *CD19*, *CD20*, *CD21*, *CD81*, *TACI (TNFRSF13B)*, *BAFF (TNFRSF13C)*, *PTEN*, *PI3KD*, *PIK3R1*, *TWEAK*, *TRNT1*, *TTC37*, *NFKB1*, *NFKB2*, *IKZF1*, *IRF2BP*, *ATP6AP1*, *ITPKB*, *PRKCD*, *LRBA*, *and ICOS*.^13^, ^32^, ^33^ However, these genetic mutations contribute to less than 20% of CVID patients. Therefore, in majority of patients with CVID genetic basis and pathogenesis remain unclear.

Bossaller et al^34^ and Grimbacher et al^35^ reported decreased proportions of CXCR5^+^CD4^+^ cT_FH_ cells in CVID patients with inducible T cell costimulator (ICOS) deficiency. Cunill et al^14^ observed increased CD4^+^CXCR5^+^cT_FH_ cells in CVID as compared with controls; however, these differences were observed only between CVID with low‐switched B cells (smB^−^) vs normal controls. Coraglia et al^36^ reported no difference in CD4^+^CXCR5^+^ cT_FH_ cells that expressed IL‐10, IL‐21, or IL‐4 between CVID with and without autoimmune diseases as compared with controls. However, they observed increased proportions of PD1^+^CCR7^+^ T_FH_ in CVID with autoimmune diseases as compared with CVID without autoimmune diseases and controls. Cunill et al^14^ when used expression of CXCR3 and CCR6 to define cT_FH_1, cT_FH_ 2, and cT_FH_17, observed increased cT_FH_1 cells, and decreased T_FH_17 cells in CVID with low‐switched memory B cells as compared with CVID with normal switched memory B cells and healthy controls. No difference was observed in T_FH_2 cells. Unger et al^37^ also observed increased T_FH_1 and decreased T_FH_17 cells in CVID patient. Increased T_FH_1 cells were observed in patients with autoimmune manifestations and strongest shift in T_FH_1 cells was observed in CVID with increased CD21^low^ B cells. Turpin et al^38^ reported higher proportions of cT_FH_1, cT_FH_17 and low cT_FH_ 2 in CVID patients than control subjects. Increased IFN‐γ‐producing T_FH_1 cells in CVID were observed in CVID with noninfectious manifestations. However, Le Coz et al^39^ did not observed increased IFNγ producing T_FH_ cells in CVID. They observed increased IL‐21 producing T_FH_ cells and imbalance in T_FH_1/T_FH_2 to T_FH_17. We observed significantly decreased cT_FH_ 2 in CVID that is in agreement with report by Turpin et al.^39^ Our observations of decreased T_FH_17 cells in CVID are in agreement with reports of Cunill et al^14^ and Unger et al.^37^ However, similar to Le Coz et al,^39^ we did not observed any significant difference in T_FH_1 cells in CVID. Our results are different from those of increased T_FH_1 cells reported by Cunill et al^14^ and Unger et al.^35^ However, we did not analyze our data in relation to switched B cells. The role of T_FH_1 cells in the pathogenesis of CVID is questionable. Desjardins et al^40^ demonstrated that an addition of exogenous IFNγ to cultures of B cells had no effect on B cells from CVID patients. We did not observed significant difference in any of subsets of cT_FH_ cells between CVID patients with and without autoimmune disease. In various autoimmune diseases including SLE, IgG4‐related diseases, Sjogren's syndrome, rheumatoid arthritis, myasthenia gravis, autoimmune thyroid disease, different patterns in cT_FH_ cell subsets have been reported (reviewed in Ueno^31^]. Therefore, type of autoimmune diseases associated with CVID as well difference in characterization of CVID may explain discrepancy among various studies. Furthermore, we need to consider a role of regulatory lymphocytes in autoimmunity associated with CVID. We have reported decreased proportion of CD4^+^ Treg, CD8^+^ Treg, and Breg cells in CVID patients.^29^ More recently, cT_FR_ has been shown to regulate GC reaction at multiple levels.^41^, ^43^ cT_FR_ regulate proliferation and cytokine production, as well as B cell proliferation and immunoglobulin production.^43^, ^45^ Cunill et al^14^ reported decreased cT_FR_ cells in patients with CVID with low proportions of switched memory B cells. Borte et al^46^ did not observe any defect in IL‐21 or IL‐21R expression or mutations in *IL‐21* gene in CVID. However, they demonstrated that a combination of IL‐21, IL‐4, and anti‐CD40 induced class‐witched recombination and differentiation of B cells to immunoglobulin secreting cells in CVID. IL‐21R/IL‐4 double deficient mice exhibit a CVID phenotype with low IgG and IgA and normal IgM, suggesting a critical role of IL‐21, that is produced by cT_FH_ cells, in regulating immunoglobulin isotype switch.^47^

In summary, a decreased in T_FH_ cell subsets may play a role in poor GC reactions including decreased isotype switching, impaired affinity maturation, generation of memory B cells, and B cell differentiation to plasma cells that are characteristics of CVID. To understand the pathogenesis of defects in B cell compartment and autoimmune and inflammatory manifestations, further comprehensive studies of all phenotypic and functionally defined subsets cT_FH_ cells, including cT_FR_ in homogenously subclassified groups of CVID patients are needed.

## CONFLICT OF INTERESTS

The authors declare that there are no conflict of interests.

## AUTHOR CONTRIBUTIONS

YS performed the experiments, collected and analyzed the data, and wrote preliminary draft. SG conceived the idea, supervised YS, and edited the manuscript.
